# The psychometric properties of the Persian menopause rating scale

**DOI:** 10.1186/s12905-020-01027-0

**Published:** 2020-08-12

**Authors:** Leila Jahangiry, Robabeh Parviz, Mojgan Mirghafourvand, Maryam Khazaee-Pool, Koen Ponnet

**Affiliations:** 1 Health Education and Health Promotion Department, School of Public Health, Tabriz University of Medical Sciences, Tabriz, Iran; 2 Health services management research center, Tabriz University of Medical Sciences, Tabriz, Iran; 3 Department, Social Determinants of Health Research Center, Faculty of Nursing and Midwifery, Tabriz University of Medical Sciences, Tabriz, Iran; 4 Department of Public Health, School of Health, Mazandaran University of Medical Sciences, Sari, Iran; 5 Health Sciences Research Center, Addiction Research Institutes, Mazandaran University of Medical Sciences, Sari, Iran; 6 Faculty of Social Sciences, imec-mict-Ghent University, Ghent, Belgium

**Keywords:** Menopause rating scale, Health-related quality of life, Psychometric properties, Validity, Reliability

## Abstract

**Background:**

To measure the severity of menopausal complaints and determine the pattern of menopausal symptoms, a valid and reliable instrument is needed in women’s healthcare. The Menopause Rating Scale (MRS) is one of the best-known tools in response to the lack of standardized scales. The purpose of this study was to examine the psychometric properties of the MRS in an Iranian example.

**Methods:**

Participants were randomly selected from women referred to healthcare centers in Miandoab, West Azerbaijan, Iran. A total of 330 questionnaires were completed (response rate of 96.9%). Two samples were considered for analysis in the validation process. An exploratory factor analysis (EFA) was conducted on the first sample (n_1_ = 165), and a confirmatory factor analysis (CFA) was done using a second study sample (n_2_ = 165). The psychometric properties process was concluded with assessment of internal consistency and test-retest reliability.

**Results:**

The EFA with Principal Component Analysis extracted three factors explaining 75.47% cumulative variance. The CFA confirmed a three-factor structure of the 11-items MRS. All fit indices proved to be satisfactory. The relative chi-square (χ2/df) was 3.686 (*p* < .001). The Root Mean Square Error of Approximation (RMSEA) of the model was .04 (90% CI = .105–.150). All comparative indices of the model, including the Comparative Fit Index, Normed Fit Index, and Relative Fit Index, were more than .80 (.90, .87, and .80, respectively). For the overall scale, Cronbach’s alpha was .931, whereas the alpha for the subscales ranged from 0.705–0.950. The intraclass correlation was .91 (95% CI = .89–.93), *p* < 0.001.

**Conclusion:**

The results of the study indicate that the Persian model of the MRS is a valid and reliable scale. As a screening tool, the Persian MRS could be used to identify the pattern of symptoms among menopausal, premenopausal, and postmenopausal women to care for and educate them on how to identify and treat the symptoms.

## Background

Menopause is defined as the time in a woman’s life when there has been no menstrual period for 12 consecutive months [1]. Although menopause is a normal and natural physiological process in a woman’s life, it can negatively affect [2, 3] the one-third of their lifetime after menopause [4]. Studies have shown the negative impacts of menopausal symptoms on health-related quality of life (HRQOL) [5–9]. Almost all women experience multiple symptoms, such as hot flushes, sweats, sleep disorders, depression, and vaginal dryness. The onset of these symptoms may be severe enough to influence their normal daily activities and cause them to medical advice and treatment [10]. Thus, health care professionals should be aware of these menopausal symptoms.

A valid and reliable method was needed in women’s healthcare to measure the severity of menopausal complaints and determine the pattern of menopause symptoms. The Menopause Rating Scale (MRS) is one of the best-known methods developed in response to the lack of a standardized scale [11]. The MRS was developed for cross-cultural comparisons of menopausal symptoms related to health-related quality of life (HRQOL) and is available in more than 10 languages. It has been adopted in Germany, Switzerland, Spain, France, Mexico/Argentina, Turkey, Brazil, Indonesia [12], China [13], and Sri Lanka [14]. The MRS scale contain 11 items (symptoms or complaints) in three dimensions: somato-vegetative, psychological, and urogenital. Response to each of the 11 symptoms ranges from 0 (no symptoms) to 4 (severe symptoms) based on the severity of the symptoms perceived by the woman completing the scale. Although the MRS has been adapted to measure menopausal symptoms in Iranian women, the MRS has not been validated among Iranian women. Because the MRS questionnaire is short and easy to use, it was seen as a useful tool in this study to examine its psychometric properties in an Iranian. By doing so, the Persian model of the MRS can be applied in both epidemiological and outcome studies. It could also provide opportunities for future studies to compare the MRS to HRQOL among Iranian women to women living in other countries.

## Methods

### Participants and study design

In this study, we used a secondary analysis of data from a more extensive study that identified the menopausal pattern among a sample of Iranian women (Miandoab, West Azerbaijan, Iran). Participants were menopausal women who were randomly selected and recruited form health centers using the SIB (an abbreviation for Persian integrated health system) of household health files from 1 September to 30 November of 2018. The SIB uses the Electronic Health Record (HER) created for all Iranian people (http://dapa.ir/en/2018/09/05/sib-integrated-health-record-system.). The SIB database was screened for menopausal women aged 45 to 65. The search criteria excluded women with a) mental and cognitive disorders, b) musculoskeletal disabilities, and c) surgical conditions. Menopausal status was defined based on the classification of stages in the Reproductive Aging Workshop (STRAW) [15], including that it had been 12 months since the last menstruation. Women were randomly selected from the original screened group and contacted by telephone to ask participate in the study and confirm eligibility. During the phone interview, the interested eligible women were invited to refer the health centers and participate in the study. A trained researcher conducted the interviews.

### The study questionnaire

Menopause Rating Scale (MRS) is an 11-item instrument consisting of three dimensions: somatic symptoms (4 items), psychological symptoms (4 items), and urogenital symptoms (3items) [11]. The somatic symptoms include hot flushes, heart discomfort, joint and muscular discomfort, and sleep problems. The psychological symptoms include depressive mood, irritability, anxiety, and physical and mental exhaustion. The urogenital symptoms include sexual problems, bladder problems, and dryness of the vagina. Possible answers were based on the severity of symptoms, using a five-point Likert scale with 0 = none, 1 = mild, 2 = moderate, 3 = severe, and 4 = very severe. The total severity ranged from a minimum of 0 to a maximum of 44 and was determined by adding the scores of the three subscales. In addition to the MRS response data, demographic information was collected and included age, education, occupation, marital status, menstruation age, menopausal age, and obstetrics history.

### Translation

Because of the potential for the questionnaire to be influenced by the cultural context in which it was administered, a backward translation was applied [16]. Two bilingual health professionals, one Persian (the Iranian language) and the other English, independently translated the English version of the MRS into Persian. Then, a member of the research team (MM) produced a consolidated version for use in the survey. If there were differences between the two translated versions, the question was resolved through discussion with the translators to obtain a provisional unified translation. In cases where there was substantial disagreement, a third independent translator was engaged for additional review. Next, two independent English translators without previous knowledge of the questionnaire reviewed and translated the survey back to English to assess the comparability with the original English version and ensure that there were no discrepancies.

### Face and content validity

As part of this study, qualitative face validity was implemented. A sample of menopausal women (*n* = 8) was asked to assess the scale and give feedback for improvement. This process led to some changes in the wording of the scale. An expert panel evaluated the provisional Persian model of the MRS. The relevance and appropriateness of items to Iranian women and their cultural context were reviewed by three professors in midwifery, three in health education, one gynecologist, and one psychologist. Consistent with other studies [17], the survey’s Content Validity Index (CVI) was evaluated by the panel using a four-point scale: 4 = very relevant, 3 = relevant with some revisions to wording, 2 = only relevant if the text is significantly revised, and 1 = irrelevant. They also suggested changes to improve the wording of each question. If a panel member rated any question less than 4, they were asked to recommend changes. According to the World Health Organization (WHO) recommendations, a CVI score greater than 0.79 confirmed content validity [15]. For the face validity and to improve clarity, the pre-final version of the questionnaire was evaluated by 10 menopausal women with the same study eligibility. In the end, no questions were deleted, meaning that the length of the Persian model of the MRS was similar to that of the original MRS.

### Sample size

To obtain an optimal sample size, a ratio of 15 respondents to one question was used [18]. The sample size was calculated by multiplying the number of questions (11) in the MRS survey by the number of respondents (15); this resulted in a sample size of 165. Two sample sizes were considered for analysis in the validation process. The survey data from the first sample (n_1_ = 165) were used for a factor analysis (EFA). The second sample (n_2_ = 165) was used for cross-validation of the confirmatory model derived from the sample *n* = 1 data. As a result, 330 eligible participants were invited to the study, and a total of 325 menopausal women completed the questionnaires (response rate of 96.9%). Table 1 summarizes the characteristics of the participants in the two samples.

**Table 1:** Descriptive characteristics of the study samples

Characteristics	EFA sample (*n* = 160) N (%)	CFA sample (*n* = 165) N (%)
**Age (years)**		
45–50	107 (66.9)	107 (64.8)
51–55	40 (25)	45 (27.3)
56–60	13 (8.1)	13 (7.9)
**Educational level**		
Illiterate	44 (27.5)	38 (23.0)
Primary	72 (45.0)	38 (23.0)
Secondary	10 (6.3)	14 (8.5)
High school	21 (13.1)	22 (13.3)
University	13 (8.1)	21 (12.7)
**Marital status**		
Married	154 (96.3)	160 (97.0)
Single/widowed/ divorced	6 (3.7)	5 (3.0)
**Number of pregnancies**		
0	7 (4.4)	3 (1.8)
1–3	52 (32.5)	65 (39.4)
≥ 4	101 (63.1)	97 (58.8)
**BMI** (Mean, SD)	30.13 (4.7)	
≤ 18.49	2 (1.3)	1 (0.6)
18.5–24.9	26 (16.3)	38 (23.0)
25–29.9	47 (29.4)	62 (37.6)
≥30	85 (53.2)	62 (37.8)

#### Statistical analysis

The analyses were performed using the statistical program SPSS for Windows version 23.0 and Amos 24.0. The Kaiser-Meyer-Olkin (KMO) measure and Bartlett’s test of sphericity were used in order to assess the sampling adequacy of the factor analysis. Any factor with an eigenvalue equal to one or above was considered significant for factor extraction. Where the loading criterion was 0.4 or more, a principal component analysis using varimax rotation was used for extraction in the factor analysis. The Comparative Fit Index (CFI), the Tucker-Lewis Index (TLI), the Root Mean Square Error of Approximation (RMSEA), and the Standardized Root Mean Square Residual (SRMSR) were applied as fit indexes. Cut-off points for inferring adequate fit indices were set at (CFI > 0.95; TLI > 0.95; Root Mean Square Error of Approximation (RMSEA), and Standardized Root Mean Square Residual (SRMSR) with acceptable values of zero to one.

## Results

### Construct validity

**Fig. 1:**
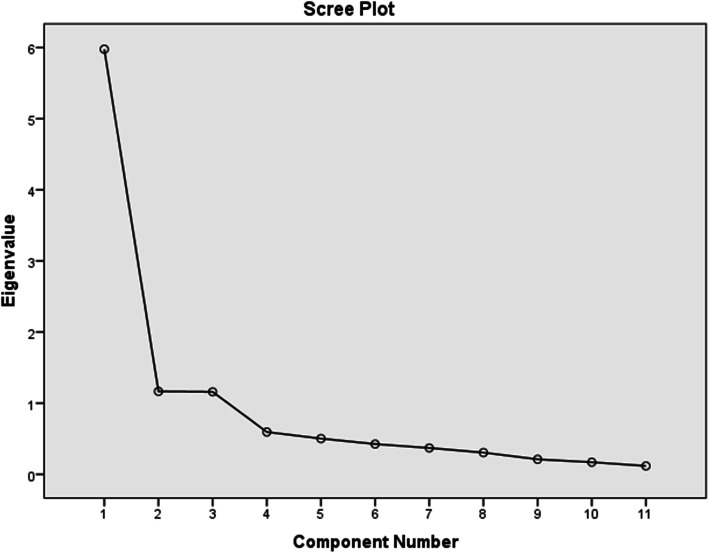
Scree plot for determining factors of the Menopause Rating Scale

The KMO was 0.855; (*P* < 0.0001), and Bartlett’s test of sphericity was significant (χ2 = 1146.18, *p* < 0.0001), indicating that the sample for the factor analysis was adequate. The principal component analysis (PCA) revealed a three-factor solution for the 11 items based on an eigenvalue greater than one. The three-factor solution explained the 75.47% variance. The scree plot also showed a three-factor solution (see Fig. 1).

All items loaded highly (> 0.50) on their respective factors except the item ‘sexual problems’, which loaded on psychological factors. Questions 1–4 (heart discomfort, joint and muscular discomfort, sleep problems, and hot flashes/sweating) correspond to the somatic symptoms saturated into a single factor (factor 1), without cross-loading items. As in the original scale, this factor was designated *somatic symptoms*. Questions 5–9 (anxiety, irritability, depressive mood, physical and mental exhaustion, and sexual problems) saturated into a single factor (factor 2) without cross-loading the questions. As in the original scale, the second factor was designated *psychological symptoms*. Questions 10 and 11 (bladder problems and dryness of vagina) saturated into a single factor (factor 3) without cross-loading the questions. As in the original scale, this factor was designated *urogenital symptoms*. Table 2 provides an overview of the factors and their factor loadings.

**Table 2:** Exploratory factor analysis of the MRS

	Items	Somatic symptoms	Psychological symptoms	Urogenital symptoms
1	Heart discomfort	0.793		
2	Joint and muscular discomfort	0.777		
3	Sleep problems	0.748		
4	Hot flushes, sweating	0.713		
5	Anxiety		0.877	
6	Irritability		0.860	
7	Depressive mood		0.766	
8	Physical and mental exhaustion		0.656	
9	Sexual problems		0.501	
10	Bladder problems			0.880
11	Dryness of vagina			0.880

*Extraction Method: Principal Component Analysis*

*Rotation Method: Varimax with Kaiser Normalization*

**Fig. 2:**
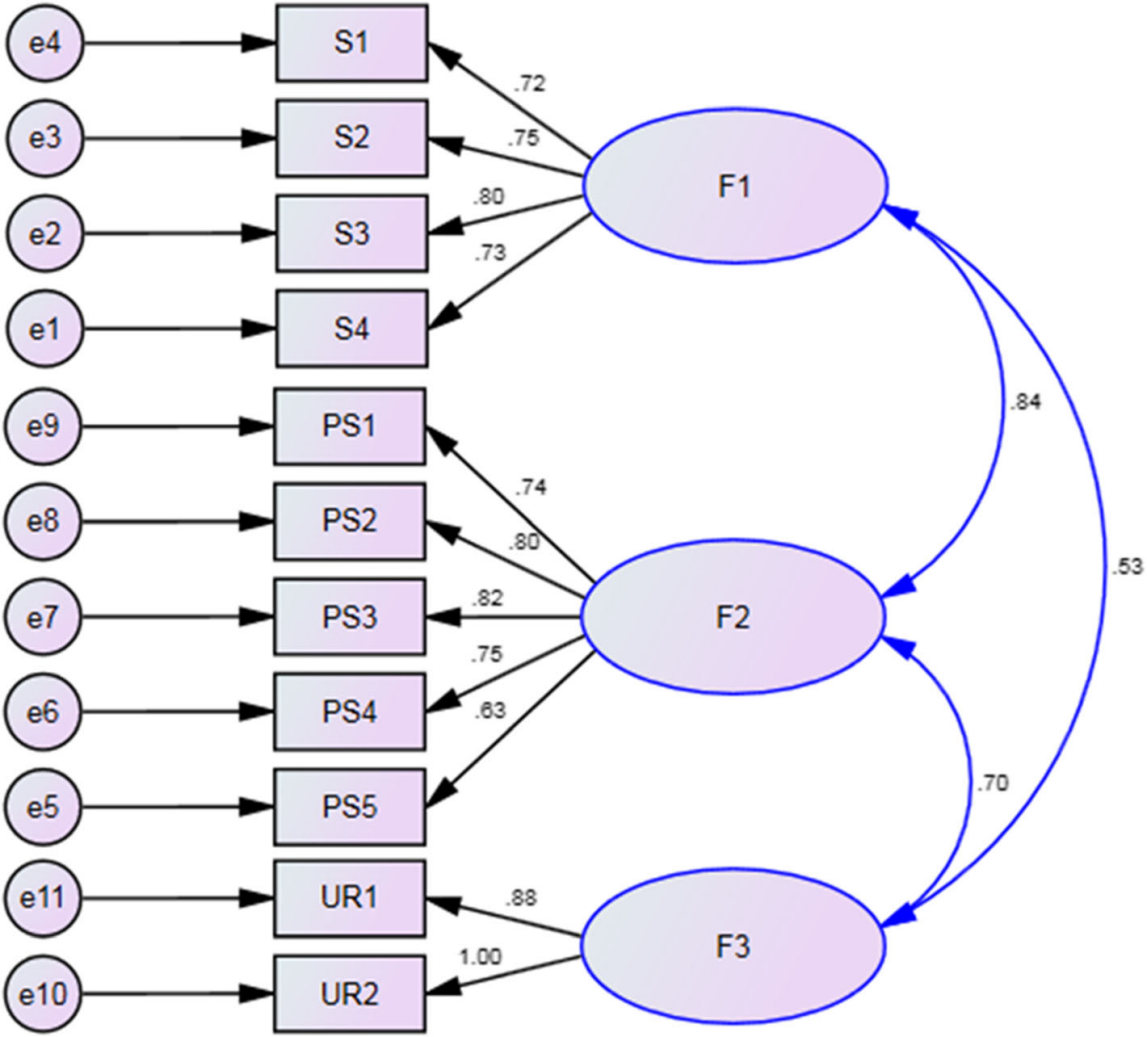
A three-factor model for the scale gained from the confirmatory factor analysis (*n* = 160)

To assess the fitness of the model obtained from the EFA, the CFA was conducted on the 11 questions in the Persian MRS. Figure 2 shows the fit of the model. Fit indices were calculated using covariance matrixes. All fit indices proved to be good. The relative chi-square (χ2/df) was equal to 3.686 (*p* < .001). The RMSEA of the model was .04 (90% CI = .01–.150). All comparative indices of the model, including CFI, NFI, and RFI, exceeded .80 (.90, .87, and .80, respectively).

### Reliability

To measure internal consistency, Cronbach’s alpha was calculated. The Cronbach’s alpha coefficient for the MRS was .931, indicating high internal reliability. The values for the subscales somatic, psychological, and urogenital symptoms were .84, .89, and .90, respectively.

A test-retest analysis was conducted to assess the stability of the MRS scale. The results indicated satisfactory results. The intra-class correlation (ICC) was .91 (95% CI = .89–.93). The ICC for somatic symptoms subscales was .84 (95% CI = .79–.87), (ICC = .89, 95% CI = .86–.91) for psychological symptoms, and .90 (95% CI = .86–.92) for urogenital symptoms.

## Discussion

This study’s goal was to evaluate the psychometric properties of the Persian model of the MRS. We concluded that the Persian MRS has sound psychometric properties and that the validity and reliability are consistent with previous MRS validation studies [12–14]. Similar to the original MRS, a three-factor structure was generated utilized. The variance of the Persian model from the original MRS was 75.47%. The three-factor model accounted for 58.8% of the total variance [11]. However, the items included in the Persian model of the MRS had one exception: one urogenital symptom (sexual problems) saturated with psychological symptoms. Accordingly, the Persian model consists of the following factors: 1 (somatic symptoms) including four questions, 2 (psychological symptoms) including five questions, and 3 (urogenital symptoms) including two questions. Probably due to cultural considerations, the Iranian women in our study consider sexual problems as psychological symptoms [19]. This finding is similar to a survey of menopausal symptoms among Sinhalese women [14] who believed that both physical exhaustion and mental exhaustion were psychological symptoms. In other validation studies, musculoskeletal problems were loaded in both somatic and urogenital subscales [14, 20]. In a Chinese study, sleep disorders were loaded in psychological symptoms, while bladder problems were loaded in somatic symptoms [13].

The CFA was used to examine whether the hypothesized model identified from the EFA fit the data. The CFA results supported the three-factor model of the Persian MRS, and the EFA and CFA confirmed the sound psychometric properties of the Persian model. Consistent with other studies [13, 14, 21], the internal consistency and test-retest reliability of the Persian model were good.

The demonstrated psychometric properties of the Persian model of the MRS indicate its potential for use as the preferred scale for the assessment of menopausal symptoms. The MRS is a user-friendly self-rating scale for menopausal symptoms that requires only a few minutes to complete and has sufficient potential before use in women’s healthcare.

This study has several limitations. First, the Iranian women selected for the survey were recruited in West Azerbaijan of Iran. Furthermore, using the original English version of the MRS during the translation process may have created a reactionary bias due to the cultural differences between the United States and Iran. To strengthen the credibility of the study results, we recommend further studies with women in other Iranian locations with different educational, cultural, and social backgrounds.

## Conclusion

This study concluded that the Persian MRS is a valid and reliable scale for assessing menopausal symptoms in Iranian women. It is a simple and informative instrument that can evaluate both symptoms and their severity. Finally, the Persian model of the MRS is a screening tool that can identify the pattern of symptoms among menopausal, perimenopausal, and postmenopausal women in need of care and inform them on how to identify and treat the symptoms.

## Data Availability

The data collection tools and datasets generated and/or analyzed during the current study are available from the corresponding author upon reasonable request.

## Abbreviations

SPSS: Statistical package for social science software package
MRS: Menopause Rating Scale
HRQOL: Health related quality of life
EFA: Exploratory factor analysis
CFA: Confirmatory factor analysis
ICC: Intra-class correlation coefficient
KMO: Kaiser–Meyer–Olkin test
PCA: Principal component analysis
CFI: Comparative Fit Index
NFI: Normed Fit Index
RFI: Relative Fit Index
